# *Arabidopsis thaliana Xylem Cysteine Protease 1 Gene* Regulates Xylem Bridge Reconnection and Delayed Incompatibility in *Arabidopsis*/*Nicotiana* Interfamilial Grafts

**DOI:** 10.3390/plants15131939

**Published:** 2026-06-23

**Authors:** Shuang Ji, Zhuying Deng, Huiyan Wu, Xiner Qin, Yongfeng Hu, Gongjian Zeng, Xiangling Shen

**Affiliations:** 1 Hubei Engineering Research Center for Three Gorges Regional Plant Breeding/Biotechnology Research Center, College of Biological and Pharmaceutical Sciences, China Three Gorges University, Yichang 443002, China; 2 School of Agriculture, Yangtze University, Jingzhou 434023, China

**Keywords:** *Arabidopsis thaliana*, *Nicotiana benthamiana*, *AtXCP1*, TE formation, interfamilial grafts, delayed incompatibility

## Abstract

*XYLEM CYSTEINE PROTEASE 1* (*XCP1*) is a cysteine protease that plays a critical role in xylem differentiation and tracheary element (TE) formation. Our previous study demonstrated that TE remodeling occurs at the graft union in the *Arabidopsis thaliana (At)*/*Nicotiana benthamiana (Nb)* interfamilial graft. Here, we identify that the *AtXCP1* transcript is specifically localized in TEs at the graft interface of the incompatible *At*/*Nb* interfamilial grafts, and its expression is reduced in these incompatible grafts compared to the compatible grafts. Analysis of *AtXCP1pro::*GFP reporter lines revealed that *AtXCP1* expression is rapidly induced by wounding at the graft interface in *At*/*Nb* interfamilial grafts during the early grafting stage. Notably, *AtXCP1* expression was significantly stronger in *At*/*Nb* heterografts than in *At*/*At* homografts, and GFP fluorescence was observed in the stock xylem at 7 days after grafting (DAG) in heterografts, a dynamic process absent in *At*/*At* homografts. We found that the *Atxcp1* mutant promoted the survival of *At*/*Nb* interfamilial grafts during the early grafting stage but decreased the survival after several months, indicating delayed incompatibility. Anatomical examination revealed that large cellular deposits accumulated at the graft interface in *Atxcp1*/*Nb* interfamilial grafts and exhibited abnormal TE morphology at later stages. Our findings identify *AtXCP1* as a key regulator of xylem reconnection and delayed incompatibility in *At*/*Nb* interfamilial grafts.

## 1. Introduction

Grafting is an ancient horticultural technique that allows two or more different plant parts to be combined into a single organism [1,2]. During grafting, successful grafts depend on the formation of a vascular connection between the scion and rootstock [3,4,5,6,7,8]. Indeed, intraspecific grafts are significantly more likely to be compatible than interspecific grafts [1,9,10,11,12]. Recent studies have successfully established interfamily grafting in different species [6,7,13,14]. The nightshade family, comprising the *Nicotiana* and *Petunia* genera, is grafted with diverse species [14,15]. Previous work establishes *Arabidopsis thaliana (At)*/*Nicotiana benthamiana (Nb)* interfamily grafts and identifies that tracheary element (TE) formation is critical to healing the graft junction during the interfamily grafting process [13]. However, interfamilial grafts often exhibit delayed incompatibility, which may take months or even years to manifest, after which grafts begin to fail [1,16,17,18]. Anatomical analysis identifies that distinct xylem bridges span the graft junction in compatible Solanaceae heterografts and are absent in incompatible ones [7]. Spiraling TE bundles are detected at the graft junction in incompatible interfamilial grafts, with more than 5 spirals observed in highly incompatible grafts [13,18]. Indeed, grafting is widely used in agriculture to control pests and diseases and for stress responses [19]. Watermelon shoots increased cold tolerance when grafted onto pumpkin or figleaf gourd [20]. Solanaceous crops (eggplant, tomato, potato, and sweet pepper) were used with grafting techniques to protect against root–knot nematodes (RKN, *Meloidogyne spp.*) [21]. However, interfamilial grafts often exhibit delayed incompatibility, where an early vascular connection does not guarantee long-term graft success. This highlights the need to understand the molecular mechanisms that maintain xylem continuity over time. To address this, we used an *At*/*Nb* interfamily graft as a model system due to its short life cycle, genetic amenability, and extensive mutant resources. The objective is to explore the molecular mechanism (e.g., *XYLEM CYSTEINE PROTEASE 1* (*XCP1*)) of vascular differentiation at the graft interface in delayed incompatibility. These findings can be translated into agriculturally important solanaceous crop applications. Thus, the *At*/*Nb* system provides a fast and tractable platform to discover target genes and mechanisms that may eventually be manipulated to expand the scope of interfamily grafting in agriculture.

Although TEs are xylem vessel elements that play a critical role at the graft junction, the gene regulatory pathways that control xylem formation in interfamily grafts remain poorly defined. Several regulators have been implicated, including *WUSCHEL-RELATED HOMEOBOX 4 (WOX4)*, *VASCULAR-RELATED NAC-DOMAIN 7* (*VND7*), *CELLULOSE SYNTHASE A4* (*CESA4*), and *XCP* genes. *WOX4* acts downstream of the *PHLOEM INTERCALATED WITH XYLEM (PXY)* to regulate cambial proliferation [22]. *WOX4* is detected in *Solanaceae* interfamily grafts and reveals the upregulation of *SlWOX4* during successful grafting; the *slwox4* mutant failed to form a xylem connection across the graft junction [6]. Transcriptome analysis identifies *VND7* and *CESA4* as activators of xylem tissue at the graft junction [23]. The homologous *XCP1* and *XCP2* genes encode key enzymes in the programmed cell death (PCD) of TEs and are activated by *VND6* and *VND7*, which participate in xylem vessel differentiation [24,25]. *NbXCP* genes and *NbVND7* gene have been demonstrated to regulate xylem formation that can enhance *Nicotiana* interfamily grafting [26,27]. However, the function of *AtXCP1* in *At*/*Nb* interfamily grafts remains unclear.

Our previous study revealed that TE formation is a significant key point at the graft junction in *At*/*Nb* interfamilial grafts, and spiraling TE bundles are observed at the graft interface in incompatible *At*/*Nb* interfamilial grafts [13]. *AtXCP1* encodes a cysteine protease that executes programmed cell death (PCD) during xylem vessel differentiation, which is essential for functional TE maturation [24,25]. At the graft interface, newly formed TEs must undergo proper PCD to establish a continuous xylem bridge, and the spiral-like TE bundles observed in incompatible *At*/*Nb* grafts suggest that PCD is perturbed [13]. We therefore hypothesize that *AtXCP1*-mediated PCD is required for the TE remodeling process and xylem reconnection at the graft interface. In this study, *At*/*Nb* interfamilial grafts were used as the material, and we identified *AtXCP1* as a key regulator controlling TE formation and xylem reconnection at the graft interface post-grafting. We performed in situ hybridization and generated *AtXCP1pro*::GFP reporter lines to examine the expression patterns of the *AtXCP1* gene during *At*/*Nb* interfamilial grafts. Furthermore, we established an *Atxcp1* mutant and grafted it onto *Nb* rootstock to observe TE formation at the graft junction in different stages using scanning electron microscopy (SEM), aiming to elucidate the functional role of *AtXCP1* in interfamilial graft compatibility.

## 2. Result

### 2.1. Expression of AtXCP1 in the Incompatibility AtCol-0/Nb Interfamilial Grafts

**Figure 1:**
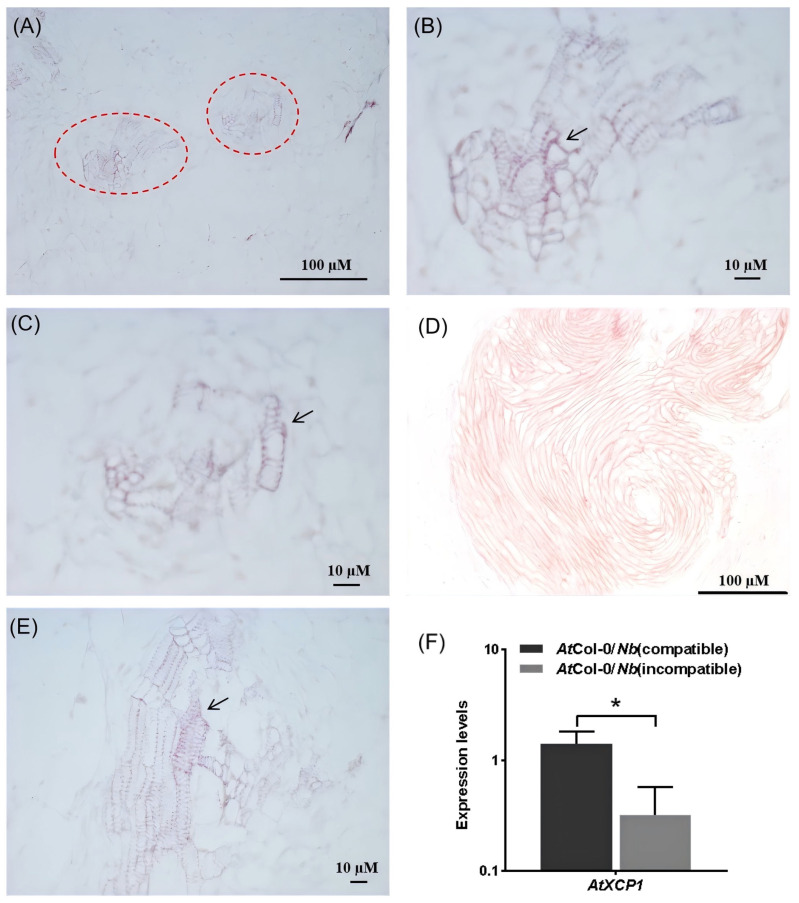
Expression of the *Arabidopsis thaliana XYLEM CYSTEINE PROTEASE 1* (*AtXCP1*) gene in incompatible *Arabidopsis thaliana (At)*/*Nicotiana benthamiana (Nb)* interfamilial graft. (**A**) Spiraling tracheary element (TE) (red dashed oval and red dashed circle) at the graft union in incompatible *At*/*Nb* interfamilial graft hybridized with *AtXCP1* antisense probe. (**B**) Spiraling TEs enlarged from (**A**) (red dashed oval). The arrow indicates the signal on the spiraling TEs. (**C**) Enlarged view of the region within the red dashed circle in (A). The arrow indicates the signal on the spiraling TEs. (**D**) Spiraling TEs at the graft union hybridized with the *AtXCP1* sense probe (negative control). (**E**) Longitudinal section of the graft union hybridized with *AtXCP1* antisense probe. The arrow indicates the signal on the TEs. (**F**) Expression levels of scions in *At*Col-0/*Nb* interfamilial grafts (* *p* < 0.05, Student’s *t*-test).

We previously constructed an *At*Col-0/*Nb* heterograft system via micrografting, which resulted in three growth phenotypes: mildly stressed, chlorotic, and retarded grafts. Among these, the retarded grafts remained quiescent and exhibited delayed incompatibility. Using the SEM technique, we previously observed extensive spiraling TE bundles at the graft union of incompatible grafts [13], and xylem bridge formation between scion and rootstock is known to be critical for graft healing [13,18]. In a previous study, the *NbXCP1* gene was identified as essential for TE formation during graft healing [26]. To investigate whether *AtXCP1* expression correlates with TE formation in the *At*Col-0/*Nb* incompatible combination, we examined its spatial expression pattern at the graft union. *AtXCP1* transcripts were detected in the spiraling TEs at the graft union (Figure 1A–D), as well as in other TEs within the graft union (Figure 1E). Furthermore, we detected the expression levels of scion in *At*Col-0/*Nb* interfamilial graft. *AtXCP1* transcript levels were significantly higher in compatible *At*Col-0/*Nb* interfamilial grafts compared to incompatible ones (Figure 1F). These expression patterns suggest that *AtXCP1* plays a role in TE formation during *At*/*Nb* interfamilial grafting.

### 2.2. AtXCP1 Is Important for Grafting and Wound Response

**Figure 2:**
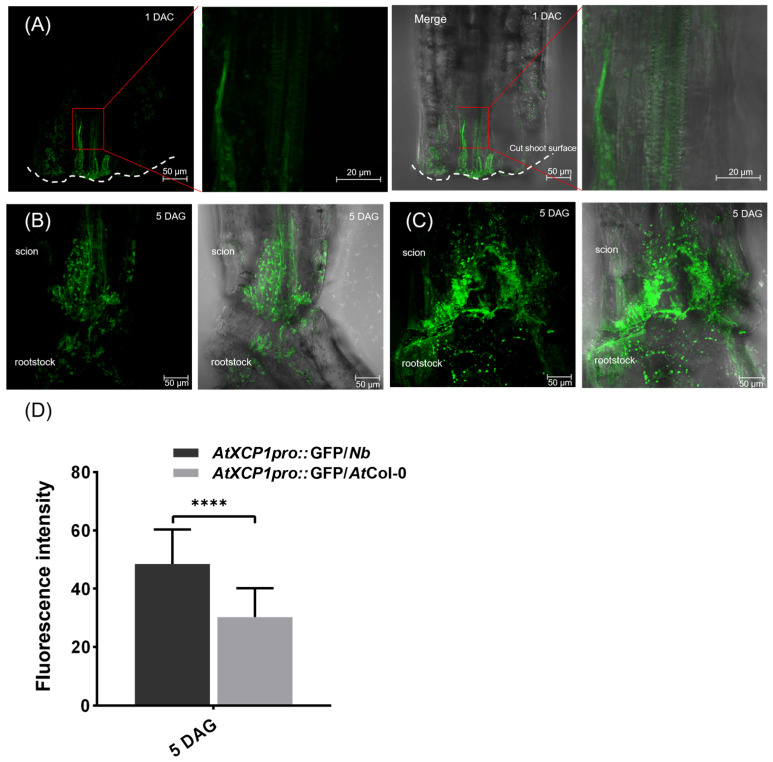
Expression of *AtXCP1pro*::GFP transgenic lines. (**A**) GFP expression at the cut surface of a hypocotyl half at 1 day after cutting(DAC). The red box indicates enlarged xylem. (**B**) GFP expression at the graft junction of an *AtXCP1pro*::GFP/*Nb* heterograft at 5 days after grafting (DAG). (**C**) GFP expression at the graft junction of an *AtXCP1pro*::GFP/*At*Col-0 homograft at 5 DAG. (**D**) Comparison of fluorescent intensity at the graft interface between *AtXCP1pro*::GFP/*Nb* heterografts and *AtXCP1pro*::GFP/*At*Col-0 homografts at 5 DAG (**** *p* < 0.0001, Student’s *t*-test).

**Figure 3:**
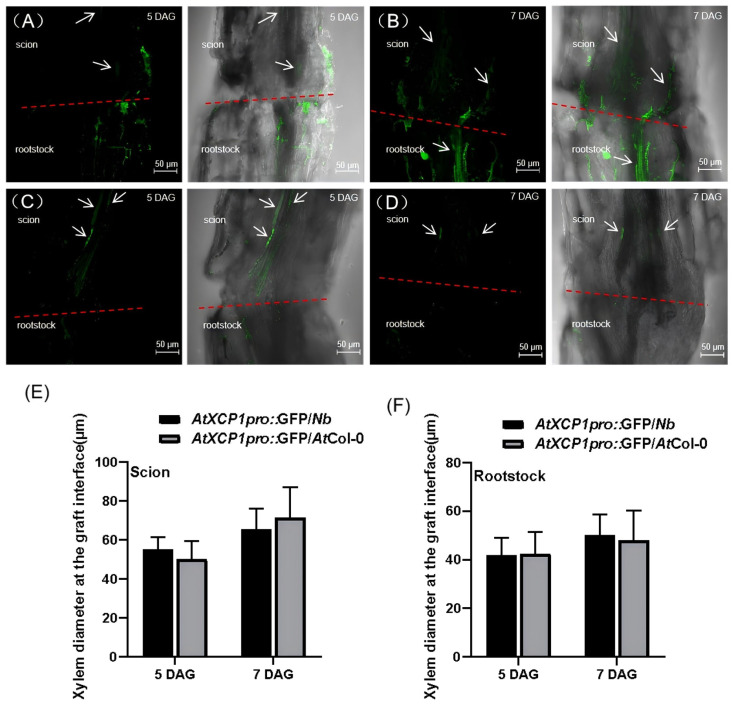
Expression dynamics of *AtXCP1pro*::GFP at the graft junction. (**A**,**B**) Longitudinal hand sections showing vascular cells (white arrows) expanding in the scion and rootstock of *AtXCP1pro*::GFP/*Nb* graft at 5 DAG and 7 DAG. (**C**,**D**) Longitudinal hand sections showing vascular cells (white arrows) expanding in the scion and rootstock of *AtXCP1pro*::GFP/*At*Col-0 graft at 5 DAG and 7 DAG. (**E**,**F**) Xylem diameter at the graft interface of the graft scion or rootstock at 5 DAG (**E**) and 7 DAG (**F**). Data are shown as the mean ± SD (n = 13–26).

To better understand the function of *AtXCP1* during grafting, we generated *AtXCP1pro*::GFP transgenic lines, which showed normal growth and development under standard conditions, allowing us to monitor *AtXCP1* expression dynamics. The *AtXCP1* promoter was detected in the vascular tissue (Supplemental Figure S1). When half-hypocotyls from 7-day-old seedlings were excised and cultured without grafting, strong GFP fluorescence accumulated at the wound site by 1 day after cutting (DAC), with upregulation observed specifically in xylem tissues surrounding the wound (Figure 2A). We next examined GFP fluorescence at the graft interface. *AtXCP1pro*::GFP scion was grafted onto *Nb* rootstock. At 5 days after grafting (DAG), the promoter of *AtXCP1* expression was detected across the graft interface in both *At*/*Nb* interfamilial grafts (*AtXCP1pro*::GFP/*Nb*) and *At* homografts (*AtXCP1pro*::GFP/*At*Col-0) (Figure 2B,C). Notably, the intensity of GFP fluorescence in the *AtXCP1pro*::GFP/*Nb* heterograft was stronger than that in the *AtXCP1pro*::GFP/*At*Col-0 homograft (Figure 2D). To further characterize xylem activation during grafting, we observed that fluorescence was restricted to the xylem tissues of the scion in both graft types at 5 DAG (Figure 3A,C). At 7 DAG, however, the expression of *AtXCP1* was detected not only in the xylem of the scion but also in the xylem of the stock in *AtXCP1pro*::GFP/*Nb* heterograft (Figure 3B). In contrast, the expression remained confined to the scion xylem in the *AtXCP1pro*::GFP/*At*Col-0 homograft (Figure 3C). To better analyze graft union formation, we evaluated the xylem diameter. No significant differences were observed between scions and rootstocks in either heterografts or homografts (Figure 3D,E). Together, these results suggest that *AtXCP1* is rapidly induced upon wounding and grafting, with its expression pattern suggesting a role in wound healing and graft regeneration.

### 2.3. Atxcp1 Mutant Regulated the Growth of At/Nb Interfamilial Grafts

**Figure 4:**
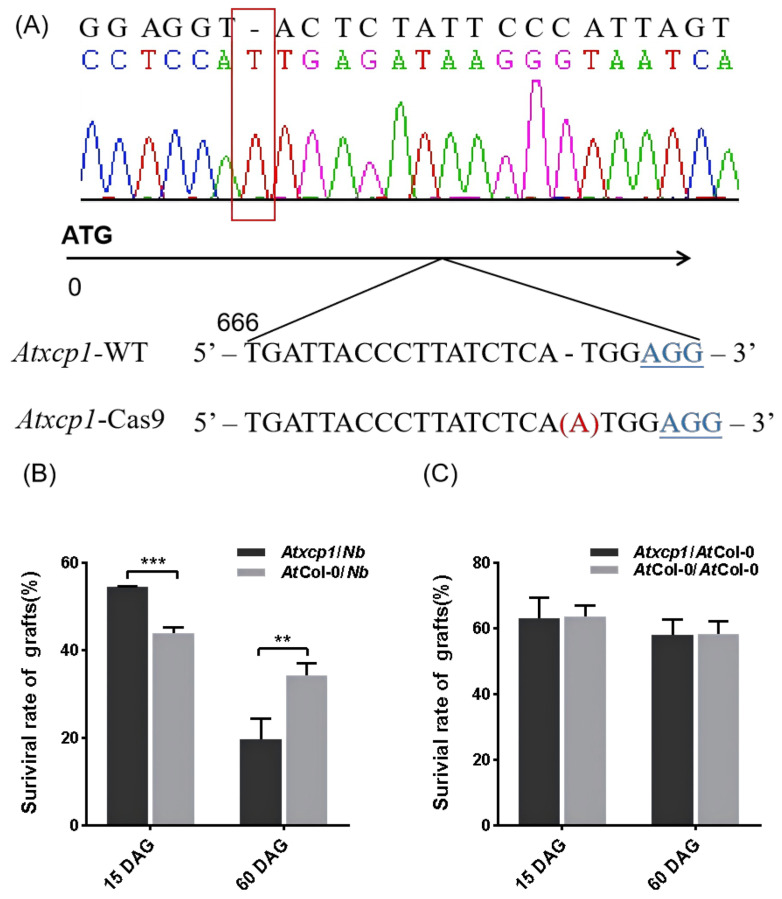
*Atxcp1* influences the growth of *At*/*Nb* interfamilial grafts. (**A**) Sequencing result showing a mutated allele of *AtXCP1* with a single adenine insertion at the codon corresponding to the 244th amino acid, induced by CRISPR-Cas9 genome editing. (**B**,**C**) Percentage of survival grafts at 15 DAG (**B**) and 60 DAG (**C**). Data are shown as the mean ± SD (n = 14–34 grafts for each time point). *p*-value was calculated by Student’s *t*-test. *** indicates *p* < 0.001. ** indicates *p* < 0.01.

To better understand the function of *AtXCP*1 during grafting, we generated an *Atxcp1* knockout mutant using a CRISPR/Cas9 system. Sequencing identified a single adenine insertion at the coding position corresponding to the 244th amino acid, resulting in a frameshift mutation and a subsequent premature stop codon. This modification confirmed the generation of the *Atxcp1* knockout mutant by targeting the *AtXCP*1 gene, which belongs to the C1 family peptidase (Figure 4A). The *Atxcp1* mutant phenotype 40 days after sowing (Supplemental Figure S2A) showed no obvious morphological differences from wild-type (*At*Col-0) plants (Supplemental Figure S2B) under normal growth conditions, consistent with previous reports that *xcp1* single mutants do not exhibit visible developmental defects [24,25]. We investigated the function of *AtXCP1* during grafting to determine whether xylem formation is important for the establishment of interfamily grafting. When *Atxcp1* scions were grafted onto *Nb* rootstocks, the survival rate of *Atxcp1*/*Nb* heterografts was significantly higher than that of *At*/*Nb* at 15 DAG. However, the survival rate of *Atxcp1*/*Nb* decreased below that of *At*/*Nb* at 60 DAG (Figure 4B). In contrast, no significant difference in survival rate was observed between *Atxcp1*/*At*Col-0 and *At*Col-0/*At*Col-0 homografts (Figure 4C). These results indicate that *AtXCP1* modulates the growth and survival of *At*/*Nb* heterografts in a time-dependent manner, promoting early survival but being required for long-term graft maintenance.

### 2.4. TE Reconnection and Cellular Deposits at the Graft Union

**Figure 5:**
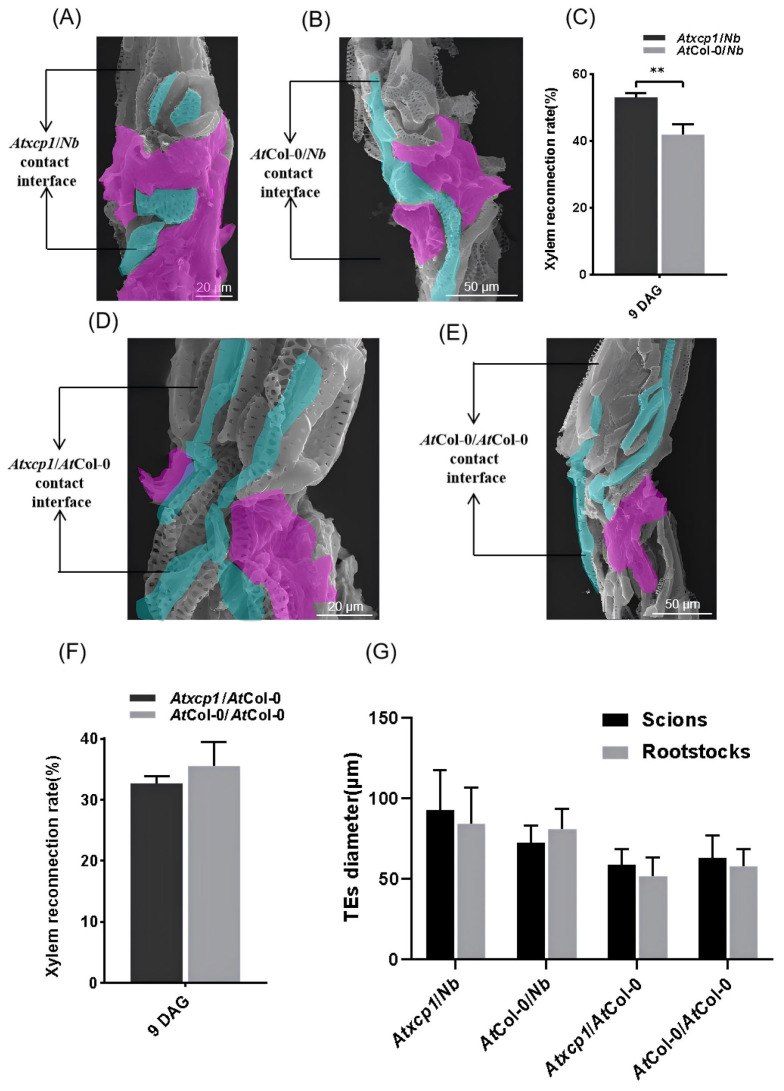
TEs structure at the graft union at 9 DAG. (**A**) *Atxcp1*/*Nb* heterograft interface. Purple indicate membrane-like substances on TEs. (**B**) *At*Col-0/*Nb* heterograft interface. Blue indicate fused TEs between the scion and rootstock. (**C**) Xylem reconnection rate in *At*/*Nb* interfamilial grafts assessed by 1% acid fuchsin staining at 9 DAG. Data are shown as the mean ± SD (n = 20–25 grafts). *p*-value was calculated by Student’s *t*-test. ** indicated *p* < 0.01. (**D**,**E**) Graft interface of *Atxcp1*/*At*Col-0 (**D**) and *At*Col-0/*At*Col-0 (**E**) homografts at 9 DAG. (**F**) Xylem reconnection rate in *At*/*At* homografts assessed by 1% acid fuchsin staining at 9 DAG. Data are shown as the mean ± SD (n = 9–18 grafts). Student’s *t*-test showed no significant difference. (**G**) TEs diameter at the graft interface in different graft combinations. Data are shown as the mean ± SD (n = 11–15 grafts). No significant differences were detected.

We previously reported that TEs form at the graft interface and contribute to xylem bridge connections in both heterografts and homografts at 9 DAG [13]. To determine whether *AtXCP1* affects xylem connection during graft union formation, we examined TEs development at 9 DAG. In both *At*/*Nb* heterografts and *At*/*At* homografts, short TEs accumulated at the graft interface and joined together to connect the xylem bridge (Figure 5A,B,D,E). Membrane-like substances were also observed covering the graft interface in both graft combinations (Figure 5A,B,D,E). However, in *Atxcp1*/*Nb* heterografts, a greater number of cellular deposits covered the graft interface, with nearly the entire interface being affected (Figure 5A). The xylem reconnection rate in *Atxcp1*/*Nb* was significantly higher than that in *At*Col-0/*Nb* grafts at 9 DAG (Figure 5C), whereas no significant differences were observed in *At*/*At* homografts (Figure 5F). We measured the connected TEs between different grafts and found that the TEs diameters were similar to each other (Figure 5G). These results indicate that *AtXCP1* influences xylem reconnection during the early stage of *At*/*Nb* heterograft formation. The observation of increased survival rate and accumulated membranous material in *Atxcp1*/*Nb* heterografts suggests that loss of *AtXCP1* may enhance early xylem bridging. This is possibly via residual cellular deposits that temporarily adhere to the graft surfaces, as loss of *Atxcp1* disrupts micro-autolysis of cellular aggregates during TE differentiation [28].

### 2.5. Large Cellular Deposits Covered the Graft Interface in Incompatibility

**Figure 6:**
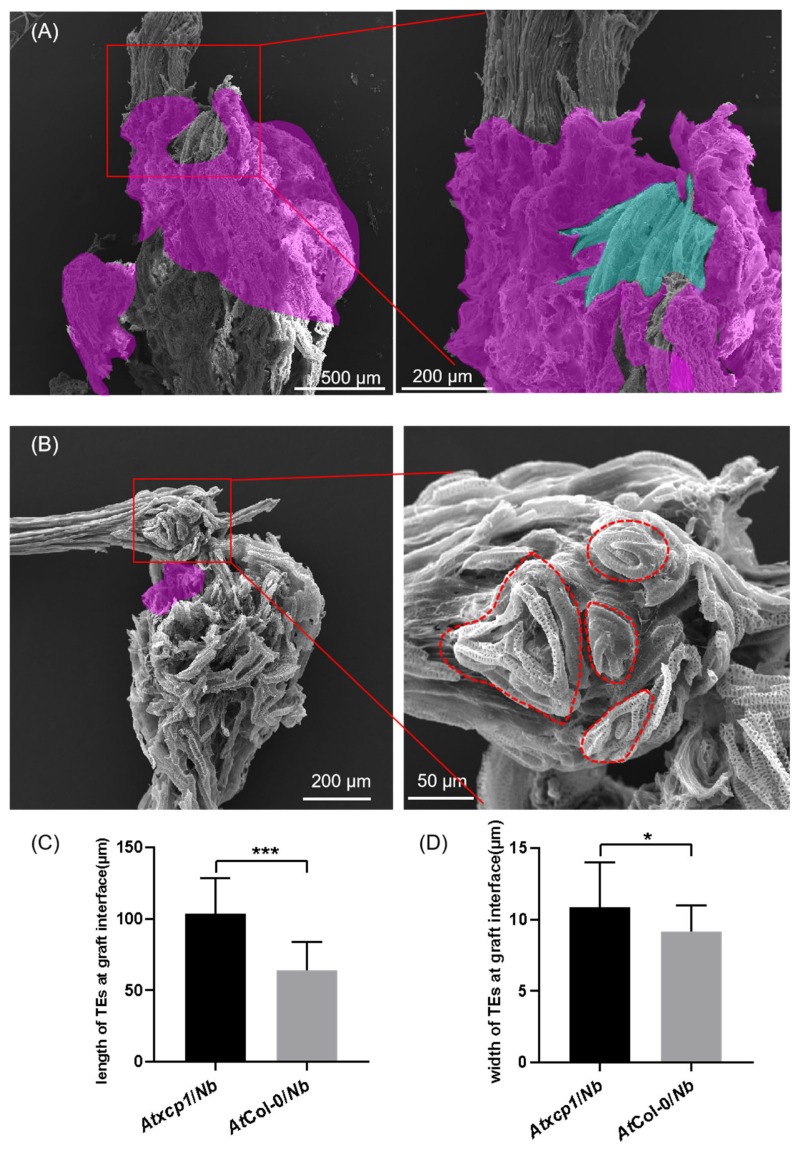
TEs structure in incompatible *At*/*Nb* heterografts. (**A**) *Atxcp1*/*Nb* graft union during the incompatible stage. Large cellular deposits (purple) covered the graft interface. **Right**: The graft interface is enlarged. Blue indicates short TEs. (**B**) *At*Col-0/*Nb* graft union during the incompatible stage. **Right**: Four spiraling TEs (red circle) are observed in the *At*Col-0 scion. (**C**) TEs length at the *At*/*Nb* interface. Data are shown as the mean ± SD (n = 30). *** indicated *p* < 0.001 (Student’s *t*-test). (**D**) TEs width at the *At*/*Nb* interface. Data are shown as the mean ± SD (n = 30). * Indicates *p* < 0.05 (Student’s *t*-test).

We continued to cultivate the grafts and monitored scion growth. All *Atxcp1*/*Nb* interfamily grafts remained quiescent (Supplemental Figure S3), a growth phenotype similar to that described for highly incompatible combinations such as *Brassica napus* (*Bn*)/*Portulaca oleracea* (*Po*) and *At*Col-0/*Nb* grafts [13,18]. It was suggested that the *Atxcp1* mutation does not overcome the inherent incompatibility between the two species. To investigate TE formation at the graft union in these incompatible grafts, we performed SEM analysis during the incompatible stage. Large cellular deposits overlapped TEs at the graft union, with only partial TEs visible in *Atxcp1*/*Nb* heterografts (Figure 6A). In contrast, *At*Col-0/*Nb* heterografts showed four spiraling TEs were observed in the scion, accompanied by numerous short TEs accumulating in the rootstock (Figure 6B). Measurements of TE dimensions at the graft interface revealed that both the length and width of TEs were increased in *Atxcp1*/*Nb* grafts compared with *At*Col-0/*Nb* grafts (Figure 6C,D). Collectively, these results suggest that *AtXCP1* regulates TE morphology and the accumulation of cellular deposits at the graft interface in *At*/*Nb* heterografts, and that these cellular features correlate with graft incompatibility.

## 3. Discussion

Understanding the xylem bridge reconnection that occurs during interfamilial graft union formation has long been a goal for plant scientists. In this study, we identified *AtXCP1* as a critical regulator of the interfamilial graft incompatibility response in *At*/*Nb* heterografts. The *AtXCP1* gene is known to be directly activated in TE formation and PCD during xylem vessel differentiation [24,25]. Our in situ analysis revealed that *AtXCP1* transcripts were present in both the spiraling TEs and TEs formation at the graft interface of incompatible *At*/*Nb* heterografts (Figure 1). This expression pattern was similar to that of *NbXCP*, which was previously shown to be critical for TE formation during graft healing [26]. However, we observed that the expression was significantly higher in *At*/*Nb* heterografts compared to *At*/*At* homografts. Moreover, fluorescence was detected in stock xylem at 7 DAG in heterografts, a spatial pattern not observed in homografts (Figure 3). The differential spatial pattern at 7 DAG could reflect either long-distance movement of GFP protein from scion to stock, or de novo activation of the *AtXCP1* promoter in stock tissues. These findings indicate that *AtXCP1* not only regulates xylem development but also specifically amplifies its response to interfamilial grafting. Additionally, the fluorescence accumulated across the wounding site and surrounding xylem, confirming that *AtXCP1* rapidly responds to mechanical damage (Figure 2A). The intensity of GFP fluorescence at the graft union of *AtXCP1pro*::GFP/*Nb* heterografts was stronger than that of *Atxcp1*/*At*Col-0 homografts at 5 DAG, suggesting that *AtXCP1* acts as a central regulator during junction formation in interfamilial grafts (Figure 2B–D). One limitation of this study is that we used a conventional GFP reporter (*AtXCP1pro*::GFP) without a non-mobile control. Thus, we cannot exclude the possibility that the fluorescence in rootstock xylem arises from long-distance protein movement rather than de novo promoter activation.

Interfamilial grafts often exhibit delayed incompatibility, which may take several months or even years post-grafting, resulting from failed vascular coordination at the graft junction [1,13,14,16,18]. In our *At*/*Nb* interfamilial grafts, the survival rate decreased significantly by 60 DAG, confirming delayed incompatibility (Figure 4B). *AtXCP* genes were known to be specifically expressed in TEs, and the *xcp1xcp2* double mutant showed normal growth under standard conditions [29]. In our study, we generated an *Atxcp1* knockout mutant and observed no unusual phenotypes compared to the wild-type. However, when *Atxcp1* scions were grafted onto *Nb* rootstocks, dynamic phenotypic changes were observed. The survival rate of *Atxcp1*/*Nb* was significantly stronger than that of *At*/*Nb* at 15 DAG, but significantly lower at 60 DAG (Figure 4B). This revealed that *AtXCP1* plays distinctly different roles at different stages after grafting. *AtXCP* genes were known to participate in micro-autolysis during xylogenesis, and *xcp1* or *xcp1xcp2* mutations cause defects in post-mortem autolysis of TEs [28,29]. This autolysis defect explains why the survival rate was significantly stronger in the early stages than in the later stages of *Atxcp1*/*Nb* heterografts. During the early stage (9–15 DAG), failure to clear cellular deposits may increase tissue adhesion across the graft junction, thereby enhancing xylem reconnection (Figure 5C) and survival (Figure 4B). However, cellular deposits accumulated at the graft junction did not represent functional vascular continuity. At 60–90 DAG, large cellular deposits accumulated across the graft interface, compromising long-distance transport and disrupting vascular function, resulting in reduced survival (Figure 4B and Figure 6A). Thus, the xylem reconnection rate in *Atxcp1*/*Nb* was significantly higher at 9 DAG despite the presence of excessive deposits (Figure 5A,C). We propose that *AtXCP1* is required not only for TE formation but for the timely autolytic clearance of TE deposits, resulting in delayed incompatibility when membranous accumulates. These observations suggest that time-dynamic analysis of graft healing is critical for assessing graft compatibility.

Furthermore, TE formation has been shown to be essential for graft establishment and subsequent scion growth [13,18,26,30]. Spiraling TE bundles were detected in incompatible interfamilial grafts [13,18]. Here, we observed that a defective *AtXCP1* induced the accumulation of large-scale membranous structures at the graft interface and inhibited the TEs formation in incompatible *Atxcp1/Nb* grafts (Figure 6). Notably, although *Atxcp1/Nb* grafts showed significantly higher survival than *At/Nb* grafts at the early stages, a substantial accumulation of cellular deposits was observed at the graft interface, resulting in the limitation of scion growth in *Atxcp1/Nb* heterografts (Figure 4, Figure 5 and Figure 6). Notably, both the length and width of TEs at the graft interface were increased in the *Atxcp1/Nb* incompatible heterograft (Figure 6C,D). Together, these results suggest that *AtXCP1* plays a key role in maintaining the balance between the synthesis and degradation of membrane-like debris at the graft interface.

## 4. Materials and Methods

### 4.1. Plant Materials, Growth Conditions, and Transformation

The wild-type *Arabidopsis thaliana* (Col-0) and the wild-type *Nicotiana benthamiana* were used in this study. To create the *Atxcp1* mutant, the target sites were selected using online tools: https://chopchop.cbu.uib.no/ (accessed on 4 May 2024). The target primers and procedure followed Wang et al. [31]. The promoter of *AtXCP1* was amplified from *At* Col-0 and then cloned into a T-DNA vector to fuse with the GFP tag. The *AtXCP1pro*::GFP transformants were selected on MS medium containing hygromycin (50 mg/mL) (Roche, Basel, Switzerland). For germination, seeds were surface-sterilized with chlorine gas for 1 h and then sown on MS medium. After stratification at 4 °C in the dark, plates were transferred to a growth room (16 h light/8 h dark, 22–23 °C) and grown vertically.

### 4.2. Grafting and Acid Fuchsin Loading

*At/Nb*, interfamilial grafting was performed according to a previously published method [13]. After 3 DAG, grafts were picked up with forceps at the scion or rootstock to test attachment [32]. If the graft junction remained attached during the manipulation, the plant was considered attached and transferred to the MS medium. The grafts were grown longitudinally in a growth chamber under long-day conditions (16 h light/8 h dark) set at 22–23 °C. For acid fuchsin staining, the cut root tip of the rootstock was submerged in a 1% (*w*/*v*) acid fuchsin solution (Sigma-Aldrich, St. Louis, MO, USA) at room temperature for 1 h. The xylem was considered reconnected if the vein stain was found in the cotyledon. All grafts were stained with acid fuchsin solution at 9 DAG, 15 DAG, and 60 DAG. For each time point, three experiments with 9–34 individual grafts were conducted.

### 4.3. Paraffin Sectioning, RNA Extraction, and In Situ Hybridization

After 60 DAG, the graft union of incompatible *At/Nb* hetero-grafted plants was fixed with 4% (*w*/*v*) paraformaldehyde (Sigma-Aldrich, St. Louis, MO, USA) in 1X PBS buffer for 12 h at 4 °C for in situ hybridization. Plant samples were then dehydrated in a graded ethanol series, substituted with Histo-Clear II (National Diagnostics, Atlanta, GA, USA), and embedded in Paraplast^®^ (Sigma-Aldrich, St. Louis, MO, USA). The samples were sectioned at a thickness of 8 µm with a rotary microtome and prepared for hybridization. Total RNA was extracted from Col-0 leaves using the TRIzol method (ThermoFisher Scientific, Waltham, MA, USA). The cDNA was synthesized from 1 µg of total RNA using a FastQuant RT Kit (TIANGEN, Beijing, China). A 1317 bp fragment of *AtXCP1* was amplified by PCR using cDNA as a template. The amplified PCR product was cloned into the pGEM^®^-T Easy Vector System (Promega, Madison, WI, USA), followed by in vitro transcription using the DIG RNA Labeling Kit (Roche, Basel, Switzerland). Nonradioactive in situ hybridization was performed according to the methods of Wong [33].

### 4.4. The Longitudinal Hand Sections and Confocal Imaging

7-day-old seedlings of *AtXCP1pro*::GFP were cut at the cotyledon and the middle of the hypocotyl region and placed on the MS medium for 1 d. The hypocotyl was cut vertically and imaged with a Leica SP8 confocal laser-scanning microscope (Leica Microsystems, Wetzlar, Germany), equipped with a 40× water immersion objective. Images were observed with the Leica LAS X software (Version 4.3). For confocal microscopy, all images were taken on a Leica SP8 laser scanning confocal microscope, equipped with a 40× water immersion objective. The graft union morphology of hetero and homo grafted plants was observed by the GFP signal with 488 nm detection. Xylem diameter quantifications of scions and rootstocks were measured with the Leica LAS X software (Version 4.3) and GraphPad 8 Software.

### 4.5. SEM Analysis

SEM was performed as described previously [13,34]. The graft union was fixed with a 4% paraformaldehyde solution for 30 min. The fixed samples were dissected under a dissecting microscope. The longitudinally dissected samples were dehydrated for 15 min each in an ethanol series of 25, 50, 75, and 100% ethanol, and dried in a 20 °C low-vacuum drier with Christ Alpha 1-4 LDplus (Osterode am Harz, Germany). The dried samples were mounted on stubs with pre-mounted carbon conductive films and coated with gold. Examination of the samples was performed with a MIRA3 field emission scanning electron microscope from TESCAN (Brno, Czech Republic). All images were analyzed using ImageJ154 software.

## 5. Conclusions

In conclusion, our study demonstrates that *AtXCP1* regulates TE formation at the graft junction in *At*/*Nb* interfamilial grafts. The specific expression pattern of *AtXCP1* indicates that it localizes to TE formation, and its expression level is significantly higher in *At*/*Nb* interfamilial grafts than in *At*/*At* homografts. Furthermore, we generated an *Atxcp1* mutant and used it as a scion grafted onto the *Nb* rootstock. We found that the survival rate of *Atxcp1*/*Nb* grafts was much higher than that of *At*Col-0/*Nb* at 15 DAG but decreased significantly by 60 DAG, confirming a delayed incompatibility phenotype. Importantly, larger cellular deposits accumulated at the graft junction in *Atxcp1*/*Nb* interfamilial grafts, providing insight into how defective autolysis leads to delayed graft failure. Together, these findings identify that *AtXCP1* is critical for xylem bridge reconnection in interfamilial grafts and reveal that its disruption leads to delayed incompatibility via aberrant membrane accumulation. Our study advances the molecular understanding of TE formation at the graft interface and provides new insights for improving interfamilial grafting success in agriculture.

## Data Availability

The original contributions presented in this study are included in the article/Supplementary Materials. Further inquiries can be directed to the corresponding authors.

## Supplementary Materials

The following supporting information can be downloaded at: https://www.mdpi.com/article/10.3390/plants15131939/s1, Figure S1: *AtXCP1pro*::GFP reporter line expression in 5-week-old plant stems; Figure S2: Atxcp1 mutant phenotype. (A) 40 days after sowing of the *Atxcp1* mutant. (B) 40 days after sowing of the AtCol-0 species; Figure S3: The phenotype *Atxcp1*/*Nb* is an incompatible interfamily graft. Right: Enlarged view of the region within the red dashed circle on the left; Table S1: Primers used in this study.
