# Supplementary material for: Arabidopsis thaliana Xylem Cysteine Protease 1 Gene Regulates Xylem Bridge Reconnection and Delayed Incompatibility in Arabidopsis/Nicotiana Interfamilial Grafts
Source: Plants (Basel). 2026 Jun 23;15(13):1939. doi: 10.3390/plants15131939 (PMC13364277; doi:10.3390/plants15131939)
Supplement: Supplementary file 1 [file plants-15-01939-s001.zip › Supplemental Table 1.pdf]

Supplemental Table S1. Primers used in this manuscript.

Name	Sequence
AtXCP1 F1	GGTGCACAGGTTTGAGGTTT
AtXCP1 R1	TGAAGCTGGATGAGCATTTG
GEMTF1	GATGTGCTGCAAGGCGATTAAG
GEMTR1	CAGCTATGACCATGATTACGC
AT-XCP1(Cas9) F1	ATATATGGTCTCGATTGGATTACCCTTATCTCATGGGTT
AT-XCP1(Cas9) R1	TGGATTACCCTTATCTCATGGGTTTTAGAGCTAGAAATAGC
AT-XCP1(GE detect) F1	CAGATCACAACAGGGAATCTGA
AT-XCP1(GE detect) R1	GATGAGCTAAAGCCTTCACCAG
AtXCP1pro F1	agttaacactagtcaagcgctAAACCTTCTATTCTCATTTGTACG
AtXCP1pro R1	ttgctcaccatggtagatctaggagcgctAGCCAAATTTGTTCACTGAGAGA
