# Supplementary figures and images for: Arabidopsis thaliana Xylem Cysteine Protease 1 Gene Regulates Xylem Bridge Reconnection and Delayed Incompatibility in Arabidopsis/Nicotiana Interfamilial Grafts
Source: Plants (Basel). 2026 Jun 23;15(13):1939. doi: 10.3390/plants15131939 (PMC13364277; doi:10.3390/plants15131939)

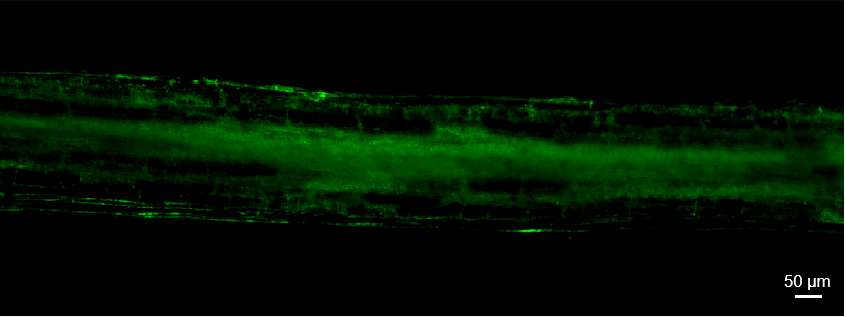

Supplement: Supplementary file 1 [file plants-15-01939-s001.zip › Supplemental Figure 1.png]

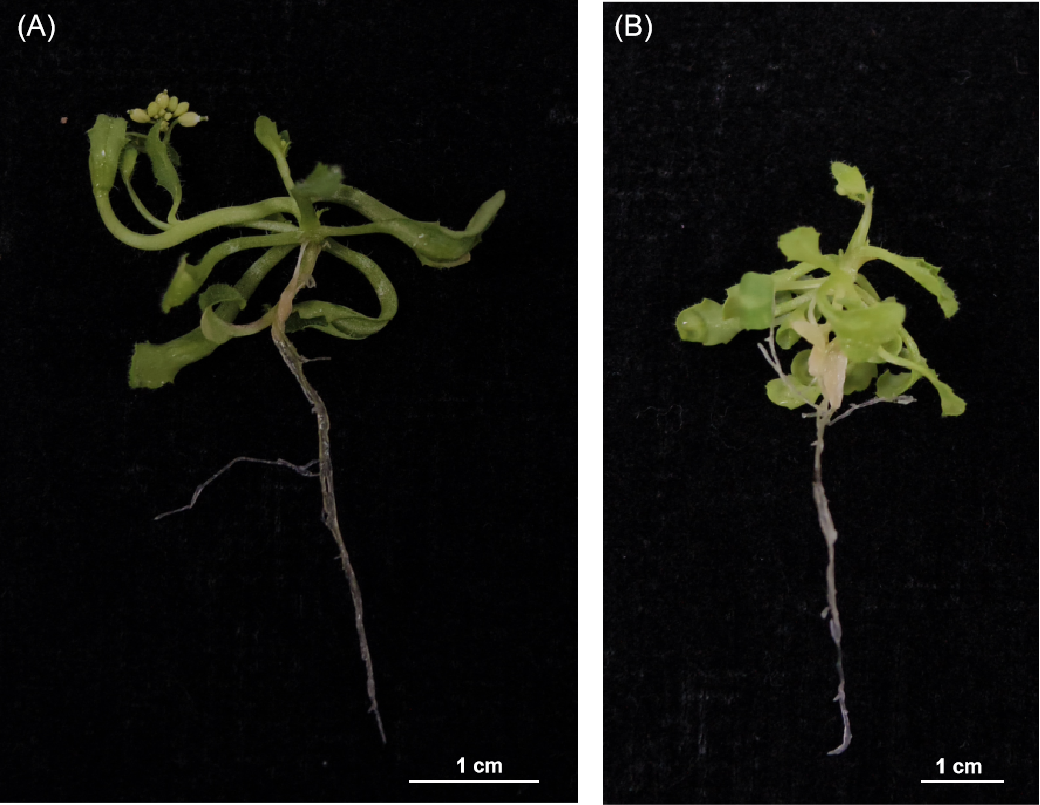

Supplement: Supplementary file 1 [file plants-15-01939-s001.zip › Supplemental Figure 2.png]

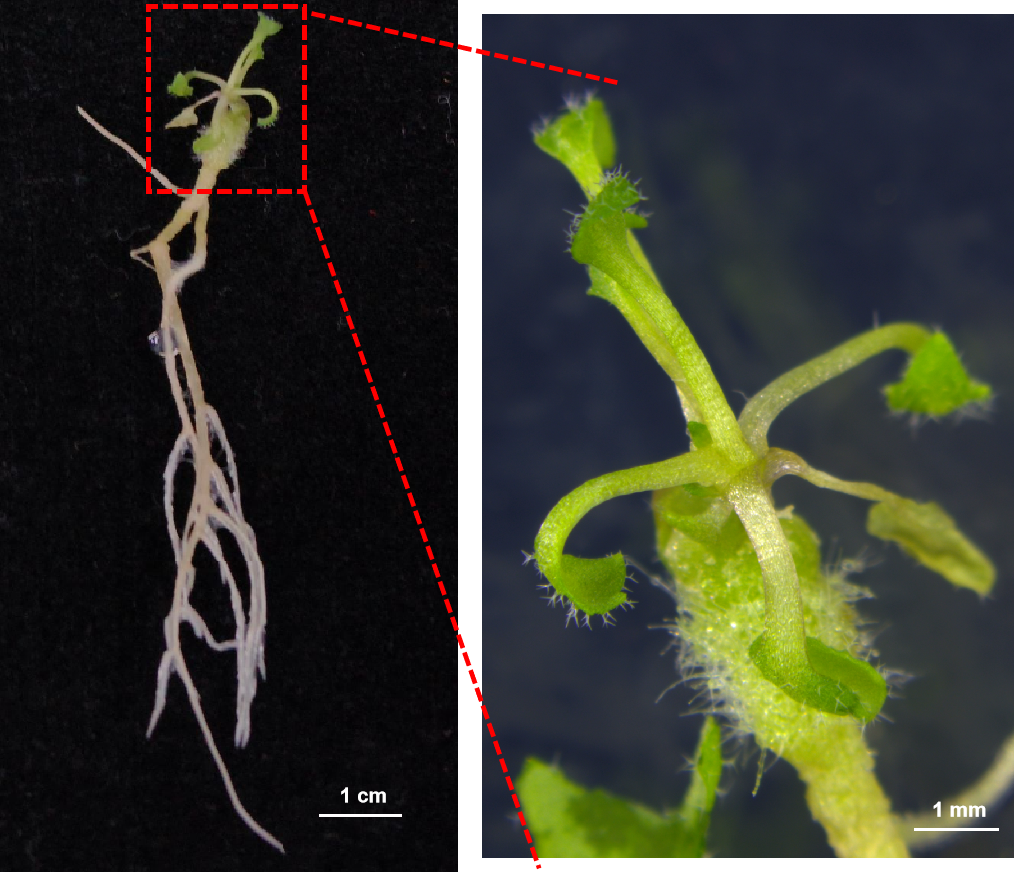

Supplement: Supplementary file 1 [file plants-15-01939-s001.zip › Supplemental Figure 3.png]
